# Genome-wide Association Study Identifies New Loci for Resistance to Sclerotinia Stem Rot in *Brassica napus*

**DOI:** 10.3389/fpls.2016.01418

**Published:** 2016-09-20

**Authors:** Jian Wu, Qing Zhao, Sheng Liu, Muhammad Shahid, Lei Lan, Guangqin Cai, Chunyu Zhang, Chuchuan Fan, Youping Wang, Yongming Zhou

**Affiliations:** ^1^National Key Laboratory of Crop Genetic Improvement, Huazhong Agricultural UniversityWuhan, China; ^2^Jiangsu Provincial Key Laboratory of Crop Genetics and Physiology, Yangzhou UniversityYangzhou, China

**Keywords:** *Brassica napus*, *Sclerotinia sclerotiorum*, genome-wide association study, transcriptomics, quantitative trait loci

## Abstract

Sclerotinia stem rot (SSR) caused by the necrotrophic fungus *Sclerotinia sclerotiorum* is a major disease in rapeseed (*Brassica napus*) worldwide. Breeding for SSR resistance in *B. napus*, as in other crops, relies only on germplasms with quantitative resistance genes. A better understanding of the genetic basis for SSR resistance in *B. napus* thus holds promise for the genetic improvement of disease resistance. In the present study, a genome-wide association study (GWAS) for SSR resistance in *B. napus* were performed using an association panel of 448 accessions genotyped with the *Brassica* 60K Infinium^®^ single-nucleotide polymorphism (SNP) array. A total of 26 SNPs corresponding to three loci, *DSRC4*, *DSRC6*, and *DSRC8* were associated with SSR resistance. Haplotype analysis showed that the three favorable alleles for SSR resistance exhibited cumulative effects. After aligning SSR resistance quantitative trait loci (QTL) identified in the present and previous studies to the *B. napus* reference genome, one locus (*DSRC6*) was found to be located within the confidence interval of a QTL identified in previous QTL mapping studies and another two loci (*DSRC4* and *DSRC8*) were considered novel loci for SSR resistance. A total of 39 candidate genes were predicted for the three loci based on the GWAS combining with the differentially expressed genes identified in previous transcriptomics analyses.

## Introduction

The white mold fungus *Sclerotinia sclerotiorum* (Lib.) de Bary is a typical necrotrophic pathogen that infects more than 400 plant species (Boland and Hall, 1994; Bolton et al., 2006), including oilseed rape (*Brassica napus* L.), the second most important oil crop worldwide (FAOSTAT, 2015). The infection of the sclerotinia pathogen to oilseed rape results in so-called sclerotinia stem rot (SSR), which can lead to serious yield losses of the crop. For example, in China, annual yield losses of 10–20% have been attributed to SSR, reaching 80% in severely infected fields. The control of SSR relies heavily on culture practices and fungicide application. However, culture practices have little effect, reflecting the wide host range of the pathogen and its capacity to survive for long periods as sclerotia. Additionally, fungicide application is not always reliable, reflecting difficulties in pinpointing the optimal time to apply fungicides. Furthermore, fungicides may cause environmental contamination and increase farming costs (Sharma et al., 2015; Derbyshire and Denton-Giles, 2016). Compared with cultural practices and fungicide application, breeding and cultivation of resistant varieties is a more efficient, economical, and environmentally friendly approach.

Unfortunately, breeding for SSR resistance is challenging, as no immune germplasm has been identified in *B. napus* or its close relatives thus far. Nevertheless, breeding practices and genetic studies have repeatedly demonstrated that resistance performance between various *B. napus* genotypes differs dramatically and that quantitative resistance is the most important form of SSR resistance breeding in *B. napus* (Li et al., 1999, 2009; Mei et al., 2011; Ge et al., 2012; Taylor et al., 2015; Wei et al., 2015). To understand the genetic basis of quantitative resistance, several studies have performed quantitative trait locus (QTL) mapping using bi-parental populations, derived normally from crosses between a partially resistant parent and a susceptible parent (Zhao and Meng, 2003; Zhao et al., 2006; Yin et al., 2010; Wu et al., 2013; Wei et al., 2014). A number of QTLs for SSR resistance have been mapped based on these studies, and conserved QTLs have been identified on chromosomes A9 and C6 through integration analyses of these QTLs based on *B. napus* genome sequences (Li et al., 2015). Despite these successes, bi-parental QTL mapping suffers from two fundamental limitations: first, only allelic diversity that segregates between the parents can be assayed, and second, the limited number of recombination events in the bi-parental population places a limit on the mapping resolution (Korte and Farlow, 2013).

As a complement to the detection of QTLs, genome-wide association studies (GWAS) have been implemented to overcome the two main limitations of bi-parental QTL mapping approaches described above. Moreover, the recent release of *B. napus* genome sequences (Chalhoub et al., 2014), together with *Brassica* single-nucleotide polymorphism (SNP) array technologies, provides an unprecedented opportunity to conduct GWAS in *B. napus*. Recently, Wei et al. (2015) conducted GWAS on SSR resistance in 347 *B. napus* accessions and identified 17 significant associations on A8 and C6, five of which were located on A8 and 12 on C6. The broad-sense heritability of stem resistance on the population was 84%, but the two loci on A8 and C6 explained only a small part of the observed phenotypic variation (Wei et al., 2015). Considering the complexity of the genetic underpinnings of SSR resistance, continuing efforts are required to identify more significant loci/genes through GWAS.

In the present study, GWAS for SSR resistance was performed in *B. napus* using an association panel with 448 accessions, which were genotyped using *Brassica* 60K Infinium^®^ SNP arrays (Liu et al., 2016). The resistance performance of the panel was investigated in two consecutive years using detached stem inoculation assays. A total of 26 SNPs corresponding to three loci were identified through GWAS. Candidate genes for three loci were predicted based on the differentially expressed genes identified through recent transcriptomics analyses (Wei et al., 2015; Wu et al., 2016). Moreover, the SSR resistance QTLs identified in the present and previous studies were compared.

## Materials and Methods

### Plant Materials

The association panel used in the present study comprised 448 rapeseed inbred lines, including 420 accessions from Asia, 18 accessions from Europe, 5 accessions from North America, and 5 accessions from Australia (**Supplementary Table S1**), selected among 521 accessions used in our recent research (Liu et al., 2016). Population structure and genetic relatedness analyses showed that most of the accessions included in the present study were distantly related (Liu et al., 2016).

### Field Experiments

All 448 accessions were grown at the experimental farm of Huazhong Agricultural University, Wuhan, China in two consecutive years (2012–2013 and 2013–2014). All field experiments followed a randomized complete block design with two replications. Each accession was grown in a plot with 10–12 plants in each of two rows, with a distance of 21 cm between plants within each row and 30 cm between rows. The field management regime followed essentially regular breeding practices.

### Assessment of Resistance to *S. sclerotiorum*

The *S. sclerotiorum* isolate SS-1 was maintained and cultured on potato dextrose agar (PDA, 25% potato, 2.5% dextrose and 1.5% agar, pH 5.8; Wu et al., 2013). The isolate was maintained at 4°C in darkness and cultured twice prior to inoculation at 23°C in darkness. Mycelial agar plugs (7 mm in diameter) punched from the margin of a 2-day-old culture of *S. sclerotiorum* grown on PDA were used as the inoculum.

Sclerotinia stem rot resistance was assessed using the detached stem inoculation assay according to Mei et al. (2012b) with some modifications. The stems of 3–6 individuals of each accession in each replicate with a length of approximately 30 cm were cut using a sharp knife at a height of 20 cm above the ground, and the cut ends were immediately wrapped with polyethylene to retain freshness. Subsequently, the detached stems were placed in plastic trays (56 × 38 × 15 cm) with wet gauze at the bottom of the trays. Each 30-cm stem was inoculated with a mycelial agar plug at two points at an interval of 10–13 cm. The inoculated stems were sprayed with a fine mist of water, and the plastic tray was covered with plastic film to maintain a high level of relative humidity. The plastic trays with inoculated stems were maintained at 22 ± 2°C in darkness. The length of lesions along the stems was measured after 5 days post inoculation (dpi). This assessment was conducted with two replications at the stage of flowering termination.

### Statistical Analysis

Broad-sense heritability was calculated as h^2^ = σ_g_^2^/(σ_g_^2^ + σ_ge_^2^/n + σ_e_^2^/nr) , where σ_g_^2^ is the genetic variance; σ_ge_^2^ is the interaction variance of the genotype with the environment; σ_e_^2^ is the error variance; n is the number of environments; and r is the number of replications. The estimates of σ_g_^2^, σ_ge_^2^, and σ_e_^2^ were obtained through ANOVA (analysis of variance) using SAS 9.3 (SAS Institute Inc, Cary, NC, USA). Best linear unbiased predictions (BLUPs) for each line across the two environments were calculated using a MIXED procedure in SAS 9.3 as phenotypes for the association analysis.

### Genome-Wide Association Analysis

The association panel was genotyped using *Brassica* 60K Illumina Infinium^®^ HD Assay SNP arrays (Clarke et al., 2016) according to the manufacturer’s protocol (Infinium^®^ HD Assay Ultra Protocol Guide)^1^ Quality control and mapping of the SNPs to *B. napus* ‘Darmor-bzh’ reference genome (Chalhoub et al., 2014) were performed and 25,573 SNPs retained for further analysis as described in our previous study (Liu et al., 2016).

Principal component analysis (PCA) based on all the 25,573 SNPs was performed using the GCTA tool (Yang et al., 2011). The relative kinship matrix of 448 *B. napus* lines was generated using the SPAGeDi software package (Hardy and Vekemans, 2002). Negative values between two accessions were set to 0. Trait-SNP association was performed using the PCA+K model implemented in TASSEL 4.0 (Bradbury et al., 2007). The significance of the associations between SNPs and the trait was based on a single threshold of *P* < 3.91 × 10^-5^ (*P* = 1/n, where n = the number of markers used; -log_10_ (1/25,573) = 4.4).

The linkage disequilibrium (LD) measurement parameter *r*^2^ was employed to estimate the LD between SNPs using the software package TASSEL 4.0 (Bradbury et al., 2007). Only homozygous SNPs were used, and heterozygous SNPs were set to missing. Marker haplotypes and LD blocks were defined for each associated locus when flanking markers showed strong LD (*r*^2^ > 0.4) with a lead SNP, as described by Hatzig et al. (2015). The interval of the LD block was considered for candidate gene disclosure.

### Alignment of SSR Resistance QTLs to the *B. napus* Genome

Quantitative trait locus for SSR resistance were collected from previous bi-parental QTL mapping studies (Zhao et al., 2006; Wu et al., 2013; Wei et al., 2014) and a (GWAS; Wei et al., 2015). All QTLs were aligned to the *B. napus* ‘Darmor-bzh’ reference genome (Chalhoub et al., 2014) based on the physical positions of markers in confidence intervals or LD blocks. The physical localization of the markers of RFLP (restricted fragment length polymorphism), SSR (single sequence repeat), and SNP (single nucleotide polymorphism) was determined using electronic PCR [e-PCR, (Schuler, 1997)] or BlastN (Altschul et al., 1997) employing the marker primer or probe sequences. For SSR markers, e-PCR was performed with the primer sequences, using the genome sequences as templates (Cai et al., 2012). The parameters for e-PCR were set to allow three mismatches and one gap for a given primer pair. If a pair of primers had multiple amplicons, we subsequently selected the amplicon for the primers according the linkage group of the QTL (Cai et al., 2012). For RFLP markers, the probe sequences were used to perform BlastN searches against the reference genome sequences with an *E*-value of 1E-20. If the probe aligned to multiple loci, we subsequently selected hits according to the linkage group of the QTL from the top several blast hits. The physical localization of the SNP markers used in the GWAS was performed by Wei et al. (2015) and Liu et al. (2016).

## Results

### *Sclerotinia* Resistance in *B. napus* Segregates Quantitatively

**FIGURE 1:**
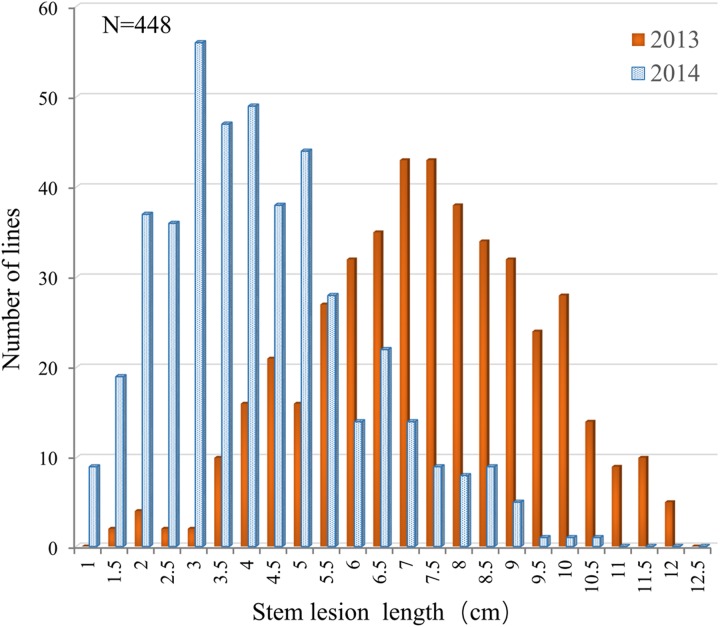
**Distribution of the lesion length on detached stems in the association panel of 448 *B. napus* accessions in 2013 and 2014**.

The resistance performance of 448 rapeseed accessions was assayed in two consecutive years (2013 and 2014) using a detached stem inoculation assay. The accessions showed relatively small variation in flowering time (153–164 days from the sowing date to flowering), which would minimize the influence of developmental spans due to differences in flowering time among genotypes on artificial inoculation assay (Mei et al., 2012a; Wei et al., 2014). Continuous and extensive phenotypic variations in *Sclerotinia* resistance were observed among the accessions. The lesion length on the stems at 5 dpi ranged from 1.25 to 13.52 cm in 2013 and from 0.52 to 10.13 cm in 2014 (**Figure 1**). Such a segregation pattern is consistent with previous observations, further implying that *Sclerotinia* resistance is a quantitative trait.

A two-way ANOVA showed that both genotype and environment have significant effects on the *Sclerotinia* resistance (*P* < 0.0001, **Table 1**), but no significant genotype-by-environment interaction was detected. The broad-sense heritability of *Sclerotinia* resistance was 61.7% (**Table 1**), consistent with the results of previous studies using the DH population (Wu et al., 2013). This result indicated that genetic variance accounted for a large portion of the phenotypic variance of *Sclerotinia* resistance in *B. napus*.

**Table 1:** Two-way ANOVA and broad-sense heritability (*h*^2^) of detached stem resistance in the 448 inbred lines of *B. napus*.

Variation	df	MS	F	P	*h*^2^ (%)
Genotype (G)	447	10.8	2.61	<0.0001	61.7
Environment (E)	1	4406.86	1064.93	<0.0001	
G × E	446	4.14	1.14	0.05	
Error	836	4.74			


### Genome-wide Association Analysis Identified Loci for *Sclerotinia* Resistance in *B. napus*

**FIGURE 2:**
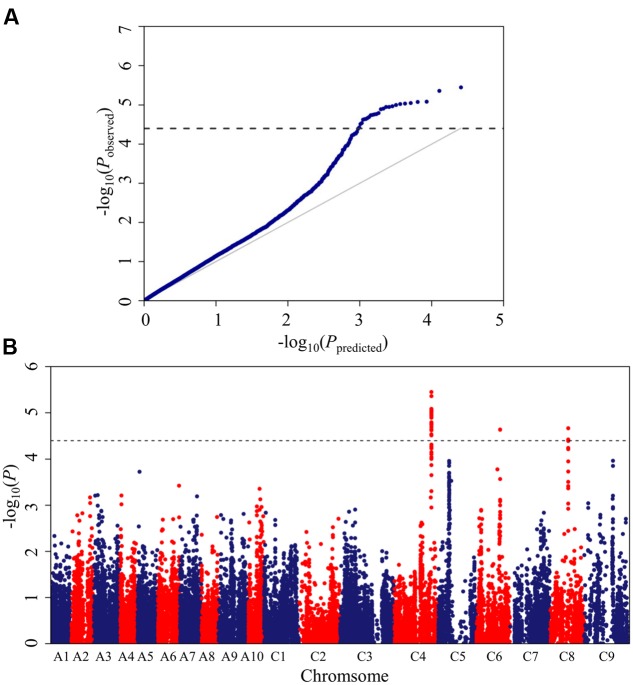
**Quantile-quantile and Manhattan plots for the GWAS (genome-wide association study) of stem resistance to *Sclerotinia sclerotiorum* in *B. napus*.** **(A)** Quantile-quantile plots for stem resistance. **(B)** Manhattan plots for stem resistance. The *dashed horizontal line* depicts the uniform significance threshold (–log_10_1/25,573 = 4.4).

The *Brassica* 60K SNP array with 52,157 SNPs was used to genotype the panel of 448 accessions. In total, 25,573 SNPs, which met the quality control criteria and had specific physical location information, were used for association mapping, as described in our previous work (Liu et al., 2016). A mixed linear model (MLM; PCA+K model) controlling population structure and kinship was selected for association mapping. This model greatly reduced false positives, as illustrated in the quantile-quantile plots (**Figure 2A**).

A total of 26 SNPs significantly associated with *Sclerotinia* resistance were identified at a threshold of *P* < 3.87 × 10^-5^ [1/25,573; –log_10_ (1/25,573) > 4.4], which corresponded to three loci (*DSRC4*, *DSRC6*, *DSRC8*) located on chromosomes C4, C6, and C8 (**Figure 2B**; **Table 2**), respectively. Among the 26 significant SNPs, 23 SNPs were located on C4, and the most significant SNP was Bn-scaff_16804_1-p674106 (–log_10_ (*P*) = 5.45), which explained 6.14% of the total phenotypic variance (**Table 2**). Two significant SNPs were detected on C8, explaining 5.26% of the phenotypic variance (**Table 2**). Only one SNP (Bn-scaff_15892_1-p326893), explaining 5.08% of the phenotypic variance, was detected on C6 (**Table 2**).

**Table 2:** Summary of SNPs (single-nucleotide polymorphism) significantly associated with stem resistance to *Sclerotinia sclerotiorum*.

Loci^a^	SNP	Chr.	Position (bp)	Allele^b^	MAF	–log_10_ (*P*)	*R*^2^ (%)^c^
*DSRC4*	Bn-scaff_16804_1-p674106	C4	41,137,804	G/T	0.21	5.45	6.14
	Bn-scaff_16804_1-p722059	C4	41,180,940	A/G	0.27	5.36	
	Bn-scaff_16804_1-p696198	C4	41,165,894	A/C	0.22	5.08	
	Bn-scaff_16804_1-p699287	C4	41,169,816	C/A	0.23	5.08	
	Bn-scaff_16804_1-p795694	C4	41,235,680	G/T	0.22	5.05	
	Bn-scaff_16804_1-p725727	C4	41,184,607	T/G	0.24	5.04	
	Bn-scaff_16804_1-p722350	C4	41,181,231	G/A	0.22	5.02	
	Bn-scaff_16804_1-p686510	C4	41,153,498	T/C	0.24	5.00	
	Bn-scaff_16804_1-p687749	C4	41,159,562	A/G	0.23	4.97	
	Bn-scaff_16804_1-p723679	C4	41,182,559	T/C	0.23	4.95	
	Bn-scaff_16804_1-p717236	C4	41,177,773	A/G	0.22	4.95	
	Bn-scaff_16804_1-p722750	C4	41,181,631	A/G	0.23	4.91	
	Bn-scaff_16804_1-p699078	C4	41,169,615	C/T	0.22	4.89	
	Bn-scaff_16804_1-p695988	C4	41,165,684	T/C	0.23	4.79	
	Bn-scaff_16804_1-p767266	C4	41,207,505	C/T	0.23	4.77	
	Bn-scaff_16804_3-p29132	C4	41,120,891	T/C	0.23	4.76	
	Bn-scaff_16804_1-p704907	C4	41,175,428	C/T	0.23	4.75	
	Bn-scaff_16804_1-p727719	C4	41,186,637	G/A	0.22	4.74	
	Bn-scaff_16804_1-p704013	C4	41,174,531	T/G	0.25	4.69	
	Bn-scaff_16804_1-p679934	C4	41,150,925	A/G	0.24	4.64	
	Bn-scaff_16804_1-p696971	C4	41,166,707	T/C	0.24	4.63	
	Bn-scaff_16804_3-p12914	C4	41,105,672	G/A	0.22	4.54	
	Bn-scaff_16804_1-p706494	C4	41,177,006	G/A	0.23	4.51	
*DSRC6*	Bn-scaff_15892_1-p326893	C6	26,238,149	A/G	0.12	4.64	5.08
*DSRC8*	Bn-scaff_16231_1-p1186822	C8	20,717,327	T/C	0.45	4.67	5.26
	Bn-scaff_16231_1-p1183915	C8	20,720,234	T/C	0.45	4.43	


*^a^QTL (quantitative trait loci) nomenclature uses the trait name followed by the chromosome number; ^b^Major allele and minor allele; the favorable alleles are underlined; ^c^Percentage of phenotypic variance explained by the lead SNP marker.*

### Alleles for *Sclerotinia* Resistance Exhibit Primarily Additive Effects in the GWAS Population

**FIGURE 3:**
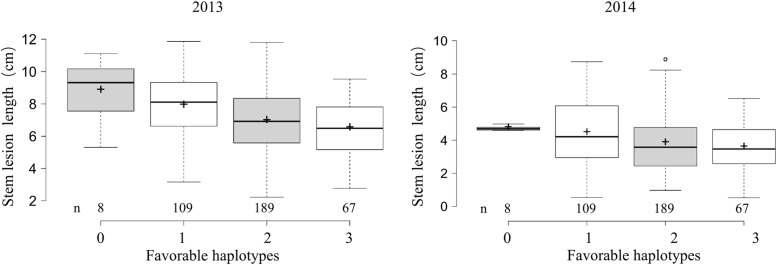
**Box plot for stem lesion length in 2 years (2013 and 2014) plotted as the favorable haplotypes.** The middle line indicates the median; the plus sign indicates the mean; the box indicates the range of the 25th to 75th percentiles of the total data; the whiskers indicate the interquartile range; and the outer dots are outliers.

To examine the effects of the identified loci on the *Sclerotinia* resistance, 373 accessions with favorable and undesirable haplotypes at ≥2 loci were categorized into four classes according to the number of favorable haplotypes. *Sclerotinia* resistance among the four groups gradually was enhanced with the increase of favorable haplotype numbers (**Figure 3**). The average lesion length in accessions with all three favorable haplotypes was significantly shorter than that of accessions with no or only one favorable haplotype, with lesion length being reduced by 24.6–26.4% (versus no favorable haplotype) and 17.7–19.6% (versus only one favorable haplotype) at 5 dpi. These results suggested that the genetic control of the *Sclerotinia* resistance in *B. napus* exhibits a largely additive effect.

### Candidate Gene Disclosure by Combining GWAS and Transcriptome Sequencing

**FIGURE 4:**
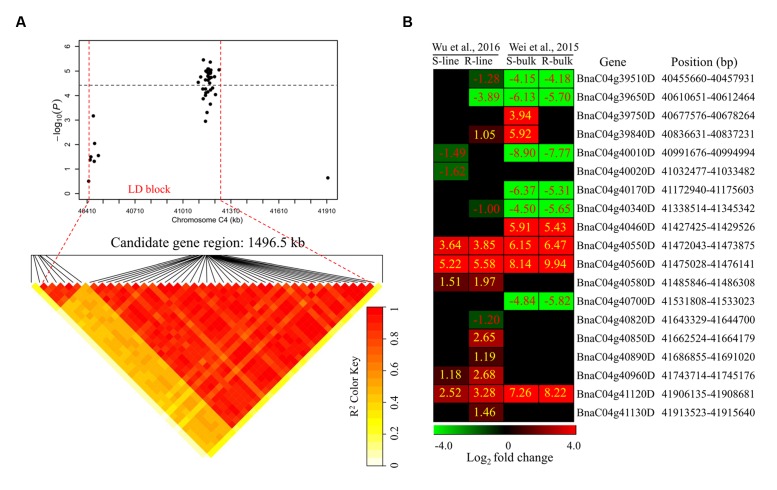
**Differentially expressed genes in the candidate gene region of *DSRC4* identified through GWAS.** **(A)** The haplotype block in strong LD (linkage disequilibrium; *r*^2^ > 0.4) with the associated SNPs (single-nucleotide polymorphism) is shown between the red dashed lines. The chromosome region of C4 (40.42–41.92 Mb) between the two flanking markers of the LD block was defined as the candidate gene region. **(B)** Heat maps of the differentially expressed genes in the candidate gene region identified through transcriptomics analyses performed by Wei et al., (2015) and Wu et al., (2016). Values labeled in the maps represent the log_2_ fold changes (inoculated/mock-inoculated) of the differentially expressed genes.

**FIGURE 5:**
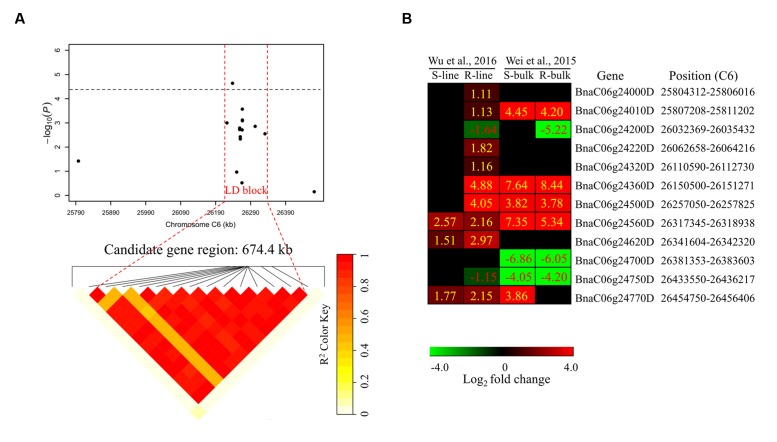
**Differentially expressed genes in the candidate gene region of *DSRC6* identified through GWAS.** **(A)** The haplotype block in strong LD (*r*^2^ > 0.4) with the associated SNPs is shown between the red dashed lines. The chromosome region of C6 (25.80–26.47 Mb) between the two flanking markers of the LD block was defined as the candidate gene region. **(B)** Heat maps of the differentially expressed genes in the candidate gene region identified through transcriptomics analyses performed by Wei et al. (2015) and Wu et al. (2016). Values labeled in the maps represent the log_2_ fold changes (inoculated/mock-inoculated) of the differentially expressed genes.

**FIGURE 6:**
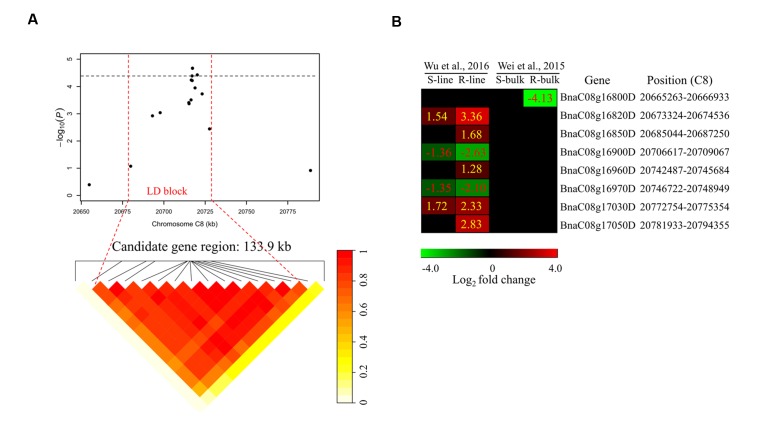
**Differentially expressed genes in the candidate gene region of *DSRC8* identified through GWAS.** **(A)** The haplotype block in strong LD (*r*^2^> 0.4) with the associated SNPs is shown between the red dashed lines. The chromosome region of C8 (20.65–20.79 Mb) between the two flanking markers of the LD block was defined as the candidate gene region. **(B)** Heat maps of the differentially expressed genes in the candidate gene region identified through transcriptomics analyses performed by Wei et al. (2015) and Wu et al. (2016). Values labeled in the maps represent the log_2_ fold changes (inoculated/mock-inoculated) of the differentially expressed genes.

To determine the chromosome regions harboring potential candidate genes, LD blocks containing the associated markers were constructed at a threshold of *r*^2^ > 0.4. For *DSRC4*, the 23 significantly associated SNPs were involved in an LD block encompassing 41 SNPs (**Figure 4A**). The significant SNPs of *DSRC6* and *DSRC8* were also located in LD blocks, both of which contained 14 SNPs (**Figures 5A** and **6A**). The region between the two flanking markers of the LD block was considered for candidate gene disclosure. Thus, the candidate gene regions of the three loci were 40.42–41.92 Mb on C4 (1496.5 kb, **Figure 4A**), 25.80–26.47 Mb on C6 (674.4 kb, **Figure 5A**), and 20.65–20.79 Mb on C8 (133.9 kb, **Figure 6A**), respectively.

Subsequently, the candidate genes in the above chromosome regions were disclosed by comparing the differentially expressed genes in the targeted as revealed through transcriptomics sequencing by Wei et al. (2015) and Wu et al. (2016). A total of 39 genes were differentially expressed in the resistant (R) or susceptible (S) lines/bulks at 48 hpi in the three candidate gene regions and were identified as candidate genes (**Figures 4B**, **5B**, and **6B**). Among these 39 differentially expressed genes, 25 genes were up-regulated and 14 genes were down-regulated. Moreover, 38.5% (15/39) of these genes were differentially expressed in both transcriptomics studies (**Figures 4B**, **5B**, and **6B**).

For *DSRC4*, 11 up-regulated and 8 down-regulated genes were identified (**Figure 4B**). Some genes that might be important for *Sclerotinia* resistance were up-regulated. For example, at 334 kb from the peak SNP (Bn-scaff_16804_1-p674106), two tau class glutathione transferase (GSTU) genes [*BnaC04g40550D* (*GSTU*4) and *BnaC04g40560D* (*GSTU*3)], which are important for protecting plants against oxidative damage (Kilili et al., 2004), were strongly induced in all lines/bulks examined in the two transcriptomics studies (**Figure 4B**; **Table 3**). In addition, two cinnamate-4-hydroxylase (C4H) genes (*BnaC04g41120D* and *BnaC04g41130D*), encoding a key enzyme in the phenylpropanoid pathway, were also induced. *BnaC04g41120D* was more strongly induced in the R-line/bulk than in the S-line/bulk, and *BnaC04g41130D* was only induced in the R-line in the study by Wu et al. (2013) (**Figure 4B**; **Table 3**).

**Table 3:** List of candidate genes identified through GWAS (genome-wide association study) and transcriptome sequencing.

Loci	Candidate gene	*Arabidopsis thaliana* locus	Description
*DSRC4*	BnaC04g39510D	AT2G28110.1	Exostosin family protein
	BnaC04g39650D	AT2G28315.1	Nucleotide/sugar transporter family protein
	BnaC04g39750D	AT2G28400.1	Unknown protein
	BnaC04g39840D	AT2G28570.1	Unknown protein
	BnaC04g40010D	AT2G28780.1	Unknown protein
	BnaC04g40020D	AT2G28790.1	Pathogenesis-related thaumatin superfamily protein
	BnaC04g40170D	AT2G28950.1	Expansin A6
	BnaC04g40340D	AT2G29260.1	NAD(P)-binding Rossmann-fold superfamily protein
	BnaC04g40460D	AT2G29350.1	Senescence-associated gene 13
	BnaC04g40550D	AT2G29460.1	Glutathione *S*-transferase tau 4 (GSTU4)
	BnaC04g40560D	AT2G29470.1	Glutathione *S*-transferase tau 3 (GSTU3)
	BnaC04g40580D	AT2G29500.1	HSP20-like chaperones superfamily protein
	BnaC04g40700D	AT2G29660.1	Zinc finger (C2H2 type) family protein
	BnaC04g40820D	AT2G30040.1	MAPKKK14
	BnaC04g40850D	AT2G30050.1	Transducin family protein/WD-40 repeat family protein
	BnaC04g40890D	AT2G30110.1	Ubiquitin-activating enzyme 1
	BnaC04g40960D	AT2G30160.1	Mitochondrial substrate carrier family protein
	BnaC04g41120D	AT2G30490.1	Cinnamate-4-hydroxylase (C4H)
	BnaC04g41130D	AT2G30490.1	Cinnamate-4-hydroxylase (C4H)
*DSRC6*	BnaC06g24000D	AT4G09420.1	Disease resistance protein (TIR-NBS class)
	BnaC06g24010D	AT1G72840.2	Disease resistance protein (TIR-NBS-LRR class)
	BnaC06g24200D	AT1G72180.1	Leucine-rich receptor-like protein kinase family protein
	BnaC06g24220D	AT1G72170.1	Domain of unknown function (DUF543)
	BnaC06g24320D	AT1G72470.1	Exocyst subunit exo70 family protein D1 (EXO70D1)
	BnaC06g24360D	AT1G72360.3	Ethylene-responsive transcription factor (ERF73)
	BnaC06g24500D	AT1G70780.1	Unknown protein
	BnaC06g24560D	AT1G70700.1	TIFY domain/Divergent CCT motif family protein
	BnaC06g24620D	AT1G70600.1	Ribosomal protein L18e/L15 superfamily protein
	BnaC06g24700D	AT1G70370.2	Polygalacturonase 2 (PG2)
	BnaC06g24750D	AT1G70260.1	Nodulin MtN21/EamA-like transporter family protein
	BnaC06g24770D	AT1G70230.1	TRICHOME BIREFRINGENCE-LIKE 27 (TBL27)
*DSRC8*	BnaC08g16800D	AT1G15860.2	Domain of unknown function (DUF298)
	BnaC08g16820D	AT2G32060.2	Ribosomal protein L7Ae/L30e/S12e/Gadd45 family protein
	BnaC08g16850D	AT1G16030.1	Heat shock protein 70B (Hsp70b)
	BnaC08g16900D	AT1G16260.2	Wall-associated kinase family protein
	BnaC08g16960D	AT1G16610.1	Arginine/serine-rich 45 (SR45)
	BnaC08g16970D	AT1G16670.1	Protein kinase superfamily protein
	BnaC08g17030D	AT1G16740.1	Ribosomal protein L20
	BnaC08g17050D	AT1G16760.1	Protein kinase protein with adenine nucleotide alpha hydrolases-like domain


For *DSRC6*, nine up-regulated and three down-regulated genes were identified (**Figure 5B**). Beside the peak SNP (Bn-scaff_15892_1-p326893), a gene encoding ethylene-responsive transcription factor 73 (*ERF73*, *BnaC06g24360D*) was up-regulated 29-fold in the R-line but was not differentially expressed in the S-line in the transcriptomics study of Wu et al. (2016); and this gene was also up-regulated 346- and 200-fold in the R- and S-bulks examined in the transcriptomics study of Wei et al. (2015) (**Figure 5B**; **Table 3**).

For *DSRC8*, five up-regulated and three down-regulated genes were identified (**Figure 6B**). Seven of these differentially expressed genes were only identified in the transcriptomics study of Wu et al. (2016). Among these genes, three were only up-regulated in the R-line, including genes encoding heat shock protein 70B (*BnaC08g16850D*), arginine/serine-rich 45 (*BnaC08g16960D*) and protein kinase protein with adenine nucleotide alpha hydrolases-like domain (*BnaC08g17050D*; **Figure 6B**; **Table 3**).

### Comparative Analysis of SSR Resistance QTLs

**FIGURE 7:**
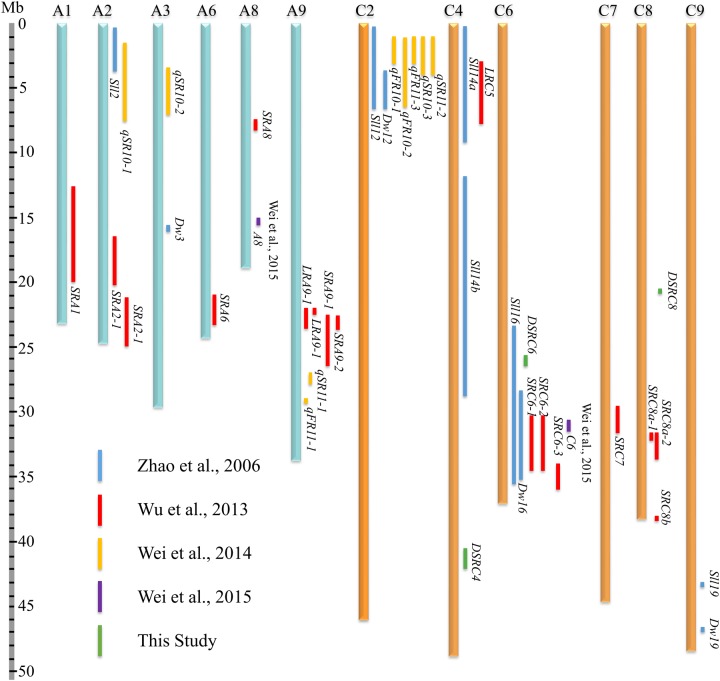
**Comparative analysis of the SSR (Sclerotinia stem rot) resistance QTLs (quantitative trait loci) identified in the present and previous studies (Zhao et al., 2006; Wu et al., 2013; Wei et al., 2014, 2015) based on the *B. napus* genome sequences**.

To compare the SSR resistance QTLs identified in the present and previous studies (Zhao et al., 2006; Wu et al., 2013; Wei et al., 2014, 2015), all QTLs were aligned to *B. napus* genome sequences based on the physical positions of markers in the confidence intervals or LD blocks. A total of 46 QTLs were collected from the present and previous studies (**Supplementary Table S2**), among which, 41 genes were successfully located to 12 *B. napus* chromosomes (A1, A2, A3, A6, A8, A9, C2, C4, C6, C7, C8, and C9; **Figure 7**; **Supplementary Table S2**). Moreover, 61% of the QTLs were mapped on the C sub-genome, suggesting that the C sub-genome harbored more loci affecting SSR resistance than the A sub-genome. In addition, the physical intervals of the QTLs identified through GWAS (an average of 0.66 Mb) were smaller than those the QTLs identified through bi-parental QTL mapping (an average of 3.66 Mb, **Figure 7**; **Supplementary Table S2**), demonstrating the higher mapping resolution of GWAS. Interestingly, *DSRC4* and *DSRC8* identified in the present study did not overlap with other QTLs, suggesting that these two loci might be new loci associated with *Sclerotinia* resistance. Three chromosomal regions with multiple QTLs were identified: A9 (22.1–29.4 Mb), C2 (0.3–6.8 Mb), and C6 (23.1–36.6 Mb, **Figure 7**). *DSRC6* identified in the present study was located in the multiple QTL region (23.1–36.6 Mb) of C6, with a physical interval that overlapped with the physical interval of *Sll16* identified by Zhao et al. (2006).

## Discussion

### Detached Stem Inoculation Assay Allows a Reliable Screening of SSR in Large Scale

Various artificial inoculation methods have been used to screen resistance against *S. sclerotiorum* in *Brassica* species. Such as petiole inoculation (Zhao et al., 2004), detached leaf inoculation (Zhao and Meng, 2003; Wu et al., 2013), oxalic acid (OA) assay (Liu et al., 1997), cotyledon inoculation (Garg et al., 2008), stem inoculation assay in the field (Zhao and Meng, 2003; Wu et al., 2013), and detached stem inoculation (Mei et al., 2012b). We used the detached stem inoculation assay in this study based on the following considerations.

The stem rot occurring at mature plant stage is the major cause of yield loss after infection of *S. sclerotiorum* in oilseed rape. For that reason, our focus on *S. sclerotiorum* resistance is to identify stem resistance QTLs mainly using stem inoculation for assessing the resistance. Detached stem inoculation was initially studied by Mei et al. (2012b), and has been successfully used to screen resistant materials (Ding et al., 2013; Mei et al., 2015), for QTL mapping (Mei et al., 2012a; Wei et al., 2014) and GWAS (Wei et al., 2015). It has been proved a stable and reliable procedure to screen stem resistance in *Brassica* species under controlled environment. On the other hand, this method is suitable for large-scale evaluation in laboratory, especially for studies using large numbers of genotypes like GWAS.

### SSR Resistance Is Determined by Minor Multiple QTLs in *B. napus*

In the present study, the broad-sense heritability of stem resistance in the 448 *B. napus* accessions was found to be 61.7% (**Table 1**), consistent with most previous studies. Zhao et al. (2006) used a petiole inoculation technique and two scoring methods [days to wilt (DW) and stem lesion length (SLL)] to evaluate the SSR resistance of two DH populations and observed that the narrow-sense heritabilities of DW and SLL were 65 and 79%, respectively, in the HUA population, and the broad-sense heritabilities of DW and SLL were 73 and 77%, respectively, in the MS population. In a previous study, we observed that the broad-sense heritabilities of leaf resistance at the seedling stage and stem resistance at the mature plant stage were 61.01 and 68.31%, respectively, in an HJ-DH population (Wu et al., 2013). Wei et al. (2014) found that the broad-sense heritabilities of field resistance and stem resistance were 63.0 and 57.2%, respectively, in a DH population with 261 lines. Wei et al. (2015) recorded the highest broad-sense heritability of stem resistance (84%) in a natural population with 347 accessions. Together, these studies demonstrated that the SSR resistance in *B. napus* shows moderate heritability (57.2–84%), and genetic variance accounts for a large portion of the observed phenotypic variance.

To dissect these complex traits, linkage analysis and association mapping are commonly used to map the genetic loci contributing to SSR resistance in *B. napus*. In the present study, we performed a GWAS for SSR resistance in *B. napus* and identified 26 significantly associated SNPs corresponding to three loci located on C4, C6, and C8, which could only collectively explain 16.48% of the total phenotypic variance (**Table 2**). These findings suggested that the power to dissect the genetic attribution for SSR resistance in a single experiment is limited. Similarly, Wei et al. (2015) detected only two SSR resistance loci located on A8 and C6 using GWAS, which seems incompatible with the observed broad-sense heritability (84%) in the study. These results might reflect the limitations of GWAS, as both rare variants and small effect sizes present problems for GWAS (Korte and Farlow, 2013). Most of the QTLs identified through bi-parental QTL mapping studies also show small effects, explaining less than 10% of the observed phenotypic variance (Zhao and Meng, 2003; Zhao et al., 2006; Yin et al., 2010; Wu et al., 2013; Wei et al., 2014). All the available studies suggest that SSR resistance in *B. napus* is a trait with very complex genetic underpinnings determined by multiple minor QTLs.

### The C Genome Harbors More Loci Affecting SSR Resistance in *B. napus*

All three loci identified in the present study were located in the C sub-genome (C4, C6, and C8. **Figure 2B**; **Table 2**). We also performed comparative analyses of SSR resistance QTLs identified in the present and previous studies. After aligning all of the collected QTLs to the *B. napus* reference genome, we observed that 61% of the mapped QTLs were located in the C sub-genome (**Figure 7**). In addition, Mei et al. (2011) observed that several wild-type varieties of *B. oleracea* possess strong resistance to *S. sclerotiorum*. These authors successfully transferred the resistance from wild-type *B. oleracea* into *B. napus* by resynthesizing *B. napus* derived from crosses between *B. oleracea* and *B. rapa* (Ding et al., 2013) or using hexaploids derived from crosses between *B. oleracea* and *B. napus* as a bridge (Mei et al., 2015). Thus, it is important to explore the genetic variations in the C genome for SSR in *B. napus*.

### High-resolution GWAS and Transcriptome Sequencing Data Offer an Opportunity to Disclose Candidate Genes for SSR Resistance

A considerable number of QTLs for SSR resistance have been identified through QTL mapping using bi-parental populations (Zhao and Meng, 2003; Zhao et al., 2006; Yin et al., 2010; Wu et al., 2013; Wei et al., 2014). Most of these QTLs were localized to large intervals (**Figure 7**; **Supplementary Table S2**), reflecting the limited number of recombination events that occur during the construction of primary mapping populations. Therefore, further application of these QTLs (such as marker-assisted selection and candidate gene prediction) are waiting for further QTL fine-mapping or map-based cloning using secondary mapping populations. There are no reports yet concerning the fine mapping or map-based cloning of SSR resistance QTLs, possibly because a large segregating population and the complexity of plant-microbe-environment interactions must be considered to accurately identify the resistance phenotype in a QTL fine-mapping study. Moreover, the small-effect QTLs and the fact that the complex amphidiploid genome increase the difficulty of fine-mapping must also be taken into consideration.

In contrast, GWAS possesses advantages over linkage analysis in terms of mapping resolution due to the exploitation of historical and evolutionary recombination (Zhu et al., 2008; Wen et al., 2016). In the present study, all of the identified SSR resistance QTLs were aligned to *B. napus* genome sequences, and the physical intervals of the QTLs identified through GWAS were indeed smaller than those identified through bi-parental QTL mapping (**Figure 7**; **Supplementary Table S2**). Because of its higher resolution, GWAS offers an opportunity to predict candidate genes. For example, Xu et al. (2015) identified 41 SNPs associated with *B. napus* flowering time, and within 300 kb of these significant SNPs, 25 candidate genes orthologous to *Arabidopsis thaliana* flowering genes were observed. Li et al. (2014) showed that two significant associations for *B. napus* erucic acid content were located 233 and 128 kb away from the key genes *BnaA.FAE1* and *BnaC.FAE1*, respectively. In the present study, the interval of the LD block containing the associated markers was taken into account for candidate gene disclosure.

Next-generation sequencing technologies enable researchers to study whole-transcriptome changes in response to *S. sclerotiorum* (Wei et al., 2015; Wu et al., 2016). In the intervals of the LD blocks of *DSRC4*, *DSRC6*, and *DSRC8*, there were 19, 12, and 8 differentially expressed genes, respectively (**Table 3**). These differentially expressed genes were treated as candidate genes, including several disease-related genes (**Table 3**; **Figures 4–6**). For example, two GSTU genes [*BnaC04g40550D* (*GSTU4*) and *BnaC04g40560D* (*GSTU3*)], located at 334 kb from the peak SNP of *DSRC4*, was found to be strongly induced after *S. sclerotiorum* infection (Wei et al., 2015; Wu et al., 2016). Williams et al. (2011) showed that *S. sclerotiorum* secreted OA suppresses host defense by manipulating the host redox environment. Initially *S. sclerotiorum* generates a reducing environment in host cells that suppress host defense responses. Once infection is established, this pathogen induces the generation of plant reactive oxygen species leading to programmed cell death of host tissue, which will in turn benefit the pathogen propagation (Williams et al., 2011). GSTUs participate in a broad network of catalytic and regulatory functions involved in the oxidative stress response (Kilili et al., 2004). Hence, *GSTU3* and *GSTU4* may be important for protecting *B. napus* against oxidative damage causing by *S. sclerotiorum* infection.

In the interval of the LD blocks of *DSRC4*, two C4H genes, *BnaC04g41120D* and *BnaC04g41130D*, may also be involved in the defense reaction against *S. sclerotiorum* infection. C4H is the second key enzyme of phenylpropanoid pathway, leading to the biosynthesis of monolignols and anthocyanins. Monolignol biosynthesis has been shown to be associated with the resistance to *S. sclerotiorum* in *Camelina sativa* (Eynck et al., 2012). The strongly induced *C4H* genes may enhance lignin deposition at the inoculated site, thus limiting the expansion of the pathogen.

Beside the peak SNP of *DSRC6*, a *ERF73* gene (*BnaC06g24360D*) was only up-regulated in the R-line in the transcriptomics study of Wu et al. (2016). *ERF73* expression was modulated by hydrogen peroxide and ethylene during hypoxia signaling in *Arabidopsis* (Yang, 2014). Hydrogen peroxide and ethylene are important signal molecule in defense against pathogens (Neill et al., 2002; Bari and Jones, 2009; Pieterse et al., 2012). So the *ERF73* may play a role downstream of hydrogen peroxide or ethylene signaling in defense response to *S. sclerotiorum* in *B. napus*. Furthermore, in the interval of the LD blocks of *DSRC6*, two genes encode disease resistance protein (TIR-NBS class) were only induced in the R-line in the study of Wu et al. (2013).

Identification of causal genes is a very complicated process. Further studies are needed to verify the molecular functions of these candidate genes through more comprehensive investigations Nevertheless, given the complexity and difficulty of map-based cloning of SSR resistance QTL, quick pinpoint of the candidate genes by combining GWAS and transcriptome sequencing might be a promising approach to dissect the quantitative genes involved in the resistance to *S. sclerotiorum* in *B. napus*.

## Author Contributions

YZ conceived the study. YZ and JW designed the experiment. JW, QZ, SL, MS, and LL performed the experiment. JW, SL, QZ, GC, CZ, CF, and YW analyzed the data. JW and YZ wrote the manuscript and all authors read and approved the final manuscript.

## Conflict of Interest Statement

The authors declare that the research was conducted in the absence of any commercial or financial relationships that could be construed as a potential conflict of interest.

## Footnotes

1 http://www.illumina.com/.
